# Manufactured Nano-Objects Confer Viral Protection against Cucurbit Chlorotic Yellows Virus (CCYV) Infecting *Nicotiana benthamiana*

**DOI:** 10.3390/microorganisms10091837

**Published:** 2022-09-14

**Authors:** Mayasar I. Al-Zaban, Sadeq K. Alhag, Anas S. Dablool, Ahmed Ezzat Ahmed, Saad Alghamdi, Baber Ali, Fatimah A. Al-Saeed, Muhammad Hamzah Saleem, Peter Poczai

**Affiliations:** 1 Department of Biology, College of Science, Princess Nourah bint Abdulrahman University, Riyadh 11671, Saudi Arabia; 2 Biology Department, College of Science and Arts, King Khalid University, Muhayil 61913, Saudi Arabia; 3 Biology Department, College of Science, Ibb University, Ibb 70270, Yemen; 4 Department of Public Health, Health Sciences College at Al-Leith, Umm Al-Qura University, Makkah 21955, Saudi Arabia; 5 Department of Biology, College of Science, King Khalid University, Abha 61413, Saudi Arabia; 6 Department of Theriogenology, Faculty of Veterinary Medicine, South Valley University, Qena 83523, Egypt; 7 Laboratory Medicine Department, Faculty of Applied Medical Sciences, Umm Al-Qura University, Makkah 24211, Saudi Arabia; 8 Department of Plant Sciences, Quaid-i-Azam University, Islamabad 45320, Pakistan; 9 College of Plant Science and Technology, Huazhong Agricultural University, Wuhan 430070, China; 10 Botany Unit, Finnish Museum of Natural History, University of Helsinki, P.O. Box 7, FI-00014 Helsinki, Finland

**Keywords:** C_60_ based-NPs, cucurbit chlorotic yellows virus, foliar application

## Abstract

Nanotechnology has emerged as a new tool to combat phytopathogens in agricultural crops. Cucurbit chlorotic yellows virus (CCYV) mainly infects Solanaceae crops and causes significant crop losses. Nanomaterials (NMs) may have efficacy against plant viruses, but the mechanisms underlying complex nanomaterials-plant-virus interactions remain elusive. We challenged *Nicotiana benthamiana* plants with GFP-tagged CCYV and observed morphological, physiological, and molecular changes in response to 21-d foliar exposure to nanoscale Fe and Zn and C_60_ fullerenes at 100 mg/L concentration for 21 days. We observed that in response to C_60_ (100 mg/L) treatment, plants displayed a normal phenotype while the viral infection was not seen until 5 days post-inoculation. On the contrary, Fe and Zn were unable to suppress viral progression. The mRNA transcriptional analysis for GFP and viral coat protein revealed that the transcripts of both genes were 5-fold reduced in response to C_60_ treatment. Evaluation of the chloroplast ultrastructure showed that NMs treatment maintained the normal chloroplast structure in the plants as compared to untreated plants. C_60_ upregulated the defense-related phytohormones (abscisic acid and salicylic acid) by 42–43%. Our results demonstrate the protective function of carbon-based NMs, with suppression of CCYV symptoms via inhibition of viral replication and systemic movement.

## 1. Introduction

Globally, over nine million souls are thought to have died and more than 800 million people are now at risk due to food security in 2020 [1]. Virus disease pandemics are a major threat to agricultural plants that are used for humankind and livestock feeding, and also, they are a major source of livelihood [2]. Plants viruses impose a serious risk to global food security by causing significant qualitative and quantitative crop losses, significantly confounding efforts to sustain food security [3]. Recently, it has been documented that globally, about 137 pests and pathogens cause qualitative and quantitative losses in the five main crops (potato, soybean, rice maize, and wheat), inducing yield losses ranging between 10.1 to 41.1% [3]. The estimated annual economic crop losses are over 30 billion USD [4] of which, 50% is contributed by the viruses of the cultivated crops that cause significant crop losses worldwide [5]. The situation becomes more complicated as the rapidly growing global population is estimated to grow by 9.8 billion people by 2050 with a 70% increased demand for food products [6]. That being said, the annual 3 billion metric tons production of agricultural crops demands the use of over 4 million tons of chemical pesticides [7]. Furthermore, the efficacy of chemical pesticides in the agricultural sector is not high, which confounds food security worldwide. As documented, about 99.9% of chemical pesticides remain off target and subsequently, they are accumulated in the environment [8]. These pesticide ingredients cause detrimental impacts by affecting soil organisms [9], soil fertility, and damaging underground water resources [10]. Furthermore, pesticides induce deleterious human health issues such as adverse birth outcomes [11], increased hypothyroidism risk [12], childhood cancer [13], hypersensitivity, allergies, and asthma [14]. 

Nanoscience stands as a new weapon in our arsenal against these mounting challenges to achieving food security [15]. Recently, nanomaterials have gained attention in the agricultural field due to their known efficacy for the management of plant diseases as compared to conventional techniques [16]. These nanomaterials (NMs) possess special characteristics enabling them to reduce inputs and maximized efficacy with reduced toxicity to the ecosystem [6]. Furthermore, NMs play a crucial role as nanofertilizers, biostimulants, nanocarriers, and antimicrobial agents [15,16]. Recently, Farooq et al. documented a comprehensive description of the relevant mechanisms of action by which NPs interact with plant viruses [17].

Previously, studies reported that foliar exposure to NMs increases plant growth and augments photosynthesis under abiotic stress [18], and maintains biomass and fruit quality and yield [19]. Furthermore, studies reported that NMs enhance crop immunity by general growth improvement and inducing antiviral defense responses. For example, Yi Hao et al. have documented that carbon and metal-based NMs enhance the phytohormone levels that ultimately reduce the accumulation of turnip mosaic virus (TuMV) by 15–60% [20]. Similarly, studies reported that Fe_3_O_4_ and ZnO NMs significantly prevent the spread and proliferation of the Tobacco mosaic virus (TMV) in *Nicotiana benthamiana* [21,22]. Recently, Adeel et al., revealed that carbon-based NMs displayed a viral protective role by suppression of viral (TMV) symptoms, inhibition of systemic movement of virus, and reduced replication of virions [23]. Though NMs might display antiviral roles of variable efficiencies, the detailed mechanisms driving NMs-plant-virus interactions largely remain elusive.

Cucurbit chlorotic yellows virus (CCYV) is an economically important and emergent phytovirus that belongs to the genus *Crinivirus* in the *Closteroviridae* family of plant viruses [24]. It was initially observed to cause infection in melon plants in Japan [25]. It possesses a bipartite genome that consists of RNA1 (8607 nt) and RNA2 (8041 nt). The proteins encoded by RNA1 play a critical role in virus replication, whereas, the RNA2-encoded proteins are important for viral encapsidation, movement, and vector-mediated transmission by whitefly (*Bemisia tabaci*) [24] in a semipersistent manner [26]. Although in nature, CCYV has only been known to infect cucurbit crops (cucumber, zucchini, melon, and watermelon), the experimental host range extends to at least four plant families (*Convolvulaceae*, *Chenopodiaceae*, *Solanaceae*, and *Asteraceae*) [24]. In general, the cell-to-cell movement of plant viruses depends on the movement protein (MP) that modifies the size exclusion limit of the plasmodesmata (PD) to facilitate viral genome transport to adjacent, uninfected cells [27,28]. Similarly, other research has shown that the systemic movement of CCYV is regulated by a protein P4.9 [29]. As a result of systemic infection, plants usually develop interveinal yellowing symptoms typical of CCYV [30].

In this study, we investigated the antiviral properties of both metals- and carbon-based NMs on tobacco (*N. benthamiana*). Fullerene (C_60_), ZnO, and Fe_3_O_4_, NMs were selected due to their known effects on crop growth in terms of weight, photosynthetic pigment, and nutrient utilization [31]. *N. benthamiana* was foliar sprayed at three concentration levels (0, 100, and 200 mg/L) of NMs suspensions for 21 days. Subsequently, the GFP-tagged CCYV (CCYV: GFP) was agroinoculated to *N. benthamiana* leaves. Afterward, the viral accumulation in the NMs-treated leaves was observed using UV light. Physiological, molecular, and biochemical parameters of plants were analyzed among all treatments to quantify the CCYV load in the systemic leaves. The impact of metal-based NMs on *N. benthamiana* leaves was evaluated using transmission electron microscopy (TEM). To the best of our knowledge, this is the first evidence that revealed that nanoparticles suppress the CCYV and promote plant growth in a dose-dependent manner. The overreaching goal of our current study was to elucidate the antiviral properties of the four tested NMs at different concentrations, expose the underlying pathway from the perspective of phytohormonal variations, and systematically extend our understanding of the prospective role of manufactured nano-objects (MNOs) in plant disease management. The findings of our work add valuable information to the development of nanopesticides and other plant disease management approaches aimed at enhancing the production of a crop.

## 2. Materials and Methods

### 2.1. Characterization of Nanoparticles

**Figure 1:**
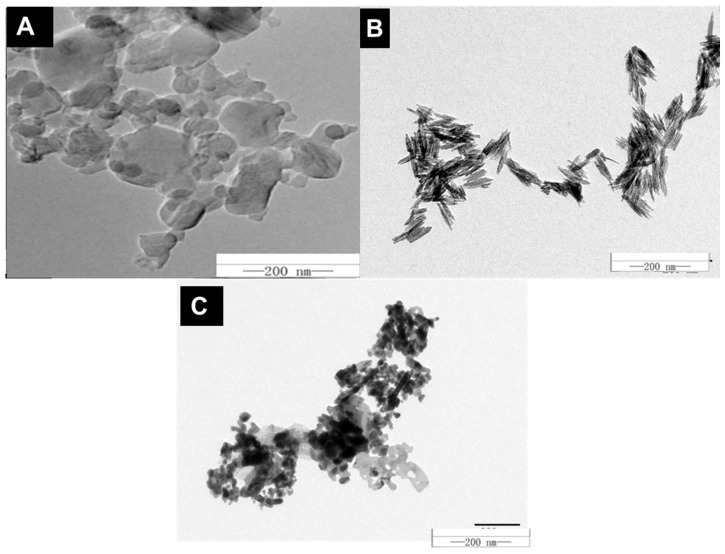
TEM images of C_60_ (**A**), Fe (**B**), and Zn (**C**). Each nanomaterial was imaged at a magnification of 107, the scale bar represents 200 nm.

All types of metal and carbon-based powdered MNOs (99.99%) were ordered from Shanghai Pantian Powder Material Co., Ltd. (Shanghai, China) and subsequently, their purification was performed before further use as described earlier [20]. An ultrasonic bath sonicator (KQ3200DE, Shumei, Cangzhou, China) was used to disperse NMs in ethanol at 40 kHz and 100 W. These suspensions were then mounted onto copper grids for size and morphology confirmation by TEM images (Hitachi H-7650, Hitachi Corp., Tokyo, Japan) observation (Figure 1). 

TEM examination revealed that spherical C_60_ exhibited aggregation with an approximated particle size of 50 ± 5; while the average diameter of Zn and Fe-based NMs remained between 20 to 30 nm (Figure 1A–C). Then, to observe the dynamic light scattering (DLS) and zeta potential, powders were dispersed in D.I. H_2_O (Table 1). The zeta potential of the C_60_, Fe, and Zn in D.I. water was −10.8 ± 0.8, 22 ± 0.7, and −14 ± 0.5 mV while DLS was 50,109 ± 500, 127 ± 3.5, and 63.92 ± 6.21 nm, respectively (Table 1). 

### 2.2. Plant Maintenance and MNOs Foliar Application

The seeds of wild-type (WT) *N. benthamiana* were germinated in a growth mixture containing black soil, perlite, vermiculite, and artificial soil (2:1:2:2) for one week at 25 °C temperature. After germination, seedlings of uniform size were transplanted into plastic pots (10 × 8 cm diameter) containing the above-mentioned substrate mixture. The plants were maintained in the controlled growth chamber using 28W T5 white fluorescent tubes (Philips, Shanghai, China), 150 µmolm^−2^s^−1^ photosynthetically active radiation (PAR), with 25–28 °C temperature and 60–65% relative humidity (RH).

Initially, we selected ten different types of NMs (CNTs, Fe, Zn, Ag, NiO, Ce, Si, Cu, rGO, and TiO_2_) based on their efficacy for plant growth and effectiveness against viral infection. Afterward, we chose their different dose levels (low, medium, and high) that didn’t induce toxicity in the plants. Based on previous studies and our preliminary experiments, we used foliar application of each NMs with one concentration of 100 mg/L [20]. About 6–8 mL of NM suspensions were foliar sprayed onto the plants for 3 weeks prior to viral treatment by agroinfiltration. This way, each *N. benthamiana* seedling was treated with about 150 mL of NMs suspension. During foliar application, black plastic was used to cover the substrate mixture to avoid direct contact with NMs. Each NM treatment included a total of 12 replications. The plants were regularly irrigated to maintain at least 60% soil moisture content. Additionally, the same volume of D.I. H_2_O without NMs was foliar sprayed on the plants used as control.

### 2.3. Agrobacterium Mediated Inoculation of CCYV

We employed a GFP-tagged viral construct (CCYV:GFP) to evaluate the effect of NMs during viral pathogenesis. Briefly, the plasmid with CCYV:GFP was chemically transformed into agrobacterium strain EHA-105 using LB with appropriate antibiotics, and the bacteria were grown overnight to reach OD600 = 1. The bacteria were then collected and resuspended in the infiltration buffer and the OD600 was adjusted to 5. Following 3 h incubation in the darkness, the agrobacterium suspension harboring CCYV:GFP was infiltrated into the lower epidermis of the *N. benthamiana* plants at the 5–6 leaf growth stage. For this purpose, a 1 mL needless syringe was used as described previously [32]. Prior to virus inoculation, the foliar spray of NMs suspension was stopped at 21 days. In order to track the CCYV infection, the plants were regularly observed for green fluorescence signals using a hand-held UV lamp and were imaged at 3, 6, and 12 dpi. The viral infection was observed in the systemic leaves exhibiting a qualitatively same growth stage. 

### 2.4. Observation of N. benthamiana Plants Using SEM and TEM

A scanning electron microscope (SEM; Hitachi-SU8020, Tokyo, Japan) was used to observe plant leaves following a previously described method [33]. In short, glutaraldehyde was used to wash the leaves followed by ethanol-mediated dehydration for 15 min. Post-dehydration, the samples were fixed using gold platinum and imaged by SEM. Additionally, a TEM (Hitachi H-7650, Hitachi Corp., Tokyo, Japan) was used to observe the ultrastructure of the chloroplasts. For this purpose, the freshly harvested leaves were washed with D.I water, exposed to glutaraldehyde (2.5%), and then treated with ethanol for dehydration. The samples were then embedded in Spurr’s resin followed by the preparation of thin sections using microtome as described earlier [34].

### 2.5. RNA Extraction, cDNA Synthesis, and RT-qPCR

The extraction of total RNA from CCYV-infected *N. benthamiana* leaves was done by using a Trizol reagent according to the manufacturer’s instructions (Invitrogen, Carlsbad, CA, USA). The quantification of RNA was done using a Nanodrop and gel electrophoresis. Subsequently, reverse transcription was performed to prepare cDNA by using HiFiScript gDNA Removal cDNA Synthesis Kit, CW2020M (Cowin Biosciences, Taizhou, China) following the manufacturer’s protocol. Afterward, qPCR was performed in triplicate by using Fast SYBR Mixture, CW0955H (Cowin Biosciences, China). The relative transcription of the target genes was calculated by the 2^−ΔΔCT^ method [35]. The details about RT-qPCR primer sequences are given in the Appendix A Table A1. 

### 2.6. Analysis of Photosynthetic Parameters and Relative Chlorophyll Contents

Photosynthesis indicators like photosynthetic rate (Pn) transpiration rate (Tr), stomatal conductance (Gs), and intercellular CO_2_ concentration (Ci) were investigated with a gas analyzer (IRGA) (Li-6400XT) (LI-COR, Lincoln, NE, USA). Chl extracts were prepared to determine the relative chlorophyll contents, using leaf tissues from virus-infected and control plants following a protocol described by Arnon [36]. A spectrophotometer was used to record the absorbance values at 645 and 663 nm wavelengths. Finally, the photosynthetic pigments were calculated by using the equation described previously [36]. 

### 2.7. Biochemical Analysis of N. benthamiana Leaves

Fresh *N. benthamiana* leaves were used for biomarker enzyme measurements. Plant hormones like abscisic acid (ABA) and salicylic acid (SA) were quantified in the NMs-treated and control plants as previously described [20]. 

### 2.8. Statistical Analysis

All analyzed data were represented as means value ± standard deviations (SD). The significant level of difference in the data sets among the various treatments was measured by two-way ANOVA followed by a *t*-test. The values of *p* < 0.05 were considered statistically significant across the treatment. 

## 3. Results

### 3.1. Impact of MNOs on the Growth of N. benthamiana

**Figure 2:**
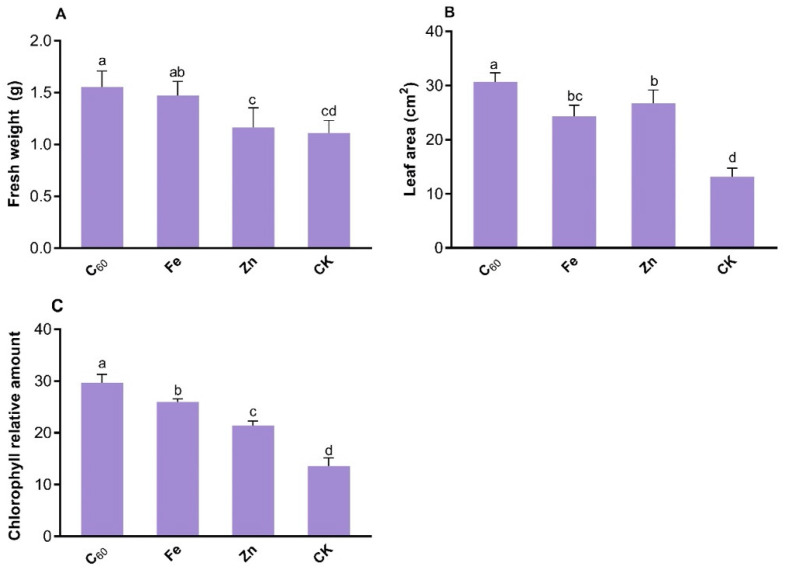
Effect of foliar application of carbon and metal-based nanoparticles (100 mg L^−1^) on *N. benthamiana*, (**A**) fresh weight, (**B**) leaf area, and (**C**) chlorophyll relative amount after the inoculation of CCYV: GFP (n = 4). Lowercase letters are Nanoparticle formula. Fullerene (C60).

To combat the pathogenic effects of CCYV: GFP on tobacco plants, carbon-based (C_60_) and metals-based (Fe and Zn) MNOs increased the plant growth such as fresh weight, leaf area, and chlorophyll contents (Figure 2). C_60_ showed a highly significant effect on fresh weight (42%), leaf area (133%), and chlorophyll relative content (118%) as compared to control plants (treated with distilled H_2_O). Additionally, C_60_ MNOs enhanced the fresh weight (6–33%), leaf area (30–48%), and chlorophyll relative content (27–61%) as compared to Fe and Zn-based MNOs at 100 mg/L foliar application (Figure 2). Moreover, Zn MNOs were unable to enhance the fresh weight of *N. benthamiana* whereas leaves area and chlorophyll content was slightly increased (though not significant) than that of control plants.

The data trend for the leaf area and chlorophyll content was homogeneous to fresh weight (Figure 2). Both carbon and metal-based MNOs significantly enhanced the plant growth under the stress of CCYV: GFP. Moreover, Fe-based MNOs significantly improved the plant growth as compared to untreated control plants, though the difference was not more than that in C_60_-based MNOs treated plant. 

Previously, numerous studies have reported that carbon and metal-based NPs have a positive impact on plant growth and development [37,38]. Carbon-based MNOs enhance plant growth by promoting nutrient absorption as well as improving photosynthesis in agricultural crops [39] and were shown to enhance the water uptake and net CO_2_ assimilation that increased the broccoli biomass [40]. Previous studies reported that remarkably high photosynthetic performance enhances plant growth and development [41]. The results of the current study are consistent with recent studies that documented that CNTs and C_60_-based NMs at 100 and 200 mg/L increased the leaf area, fresh biomass, and chlorophyll content of tobacco plants [23]. However, the impact of carbon and metal-based MNOs depends on the types of materials, size of MNOs, applied concentration, plant species, and growth conditions of the experiment. Likewise, a later study reported that Fe-based NPs (Fe_3_O_4_-NPs) enhanced the tobacco plant (that treated with TMV) growth by increasing fresh weight (52%) and dry weight (42%) as compared to the control plant (not treated with MNOs) at 100 µg/mL [21]. Another study documented that the application (foliar/ soil amended) of Fe-based MNOs increased the chlorophyll content and biomass of soybean plants [42].

García-López et al. have reported that foliar exposure of ZnO based MNOs enhanced the chlorophyll level and biomass of plants, although this increment was noted at a high concentration (1000 mg/L) in pepper plants (*Capsicum chinense Jacq*.) [43]. Whereas, in the current study, the applied concentration of Zn-based MNOs is 100 mg/L, so, Zn-based MNOs do not have highly significant effects on the tobacco plants as compared to control. 

### 3.2. Application of MNOs Activates the Plant’s Defense System by Enhancing Levels of Phytohormone

**Figure 3:**
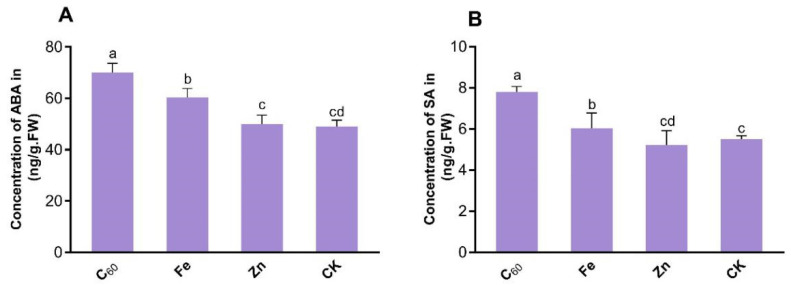
The concentration of phytohormones (ABA and SA) in the tobacco leaves treated with C_60_, Fe_3_O_4,_ and ZnO-based MNOs, (**A**) abscisic acid (ABA), and (**B**) salicylic acid (SA). *p* < 0.05, n = 3). Lowercase letters are Nanoparticle formula. Fullerene (C60).

The concentrations of two main phytohormones were examined in CCYV-infected tobacco (*N. benthamiana*) leaves upon MNOs treatment (Figure 3A,B). Exposure to 100 mg/L of C_60_ MNOs enhanced ABA levels by 42% in the virus-infected leaves as respective to control plants. Additionally, the Fe-based MNOs at 100 mg/L improved the ABA level by 23% more than that control being 19.8% less than the C_60_-based MNOs. Interestingly, the dose of Zn-based MNOs slightly increased (non-significantly) ABA only by 2% as respective to control plants (not treated with MNOs). Both C_60_ and Fe-based MNOs significantly enhanced the concentration of ABA at 100 mg/L by 42.9% and 23% as respective to CCYV control, respectively. Nevertheless, the ABA concentration was uninfluenced in response to the application of Zn-based MNOs. A similar trend was evident with SA (Figure 3B); at 100 mg/L of Zn-based MNOs have no effects on tobacco leaves SA concentration, which is similar to the results of physiological parameters. Although, the enhanced SA contents in infected leaves suggest that the application of the carbon-based MNOs at a 100 mg/L level improves defense-related results in the CCYV-infected leaves of the tobacco plant. For instance, at 100 mg/L concentration, C_60_-based MNOs increased the SA level by 41.8% as respective to control plants (Figure 3B). Moreover, exposure to the metal-based MNOs at 100 mg/L showed non-significantly enhanced SA levels in CCYV-infected tobacco leaves. Particularly, Zn-based MNOs showed a negative effect on SA concentration at 100 mg/L concentration. 

Hormones of plants are endogenous molecules that modulate plant growth and development [44]. Phytohormones also play a vital role in plant tolerance against abiotic and biotic stress. For example, ABA plays a critical role in plant physiology, including regulation of fruit ripening, seed germination, and also roles as a transitional factor to modulate aquaporin protein expression in counter to various stresses. SA is a key phytohormone that plays an important function in defense responses against biotrophic pathogens by inducing the reprogramming of antimicrobial genes [45,46,47]. Plant phytohormone signaling networks contribute an important role in growth mediating, along with resistance in plants and reaction to pathogenic infections [48]. Given that viral infection of plants regulates the development of stomata [49], ABA concentration seems to modulate the stomatal movement and consequently restrict the entry of pathogens. Increments in plant phytohormones like SA and ABA can play a crucial role in stimulating the defense system of plants against viral infection. The findings of the current study are similar to the previous study which reports that carbon-based MNOs (C_60_ and CNTs) at 100 mg/L and 200 mg/L increased the ABA (33–40%) and SA (16–37%) concentration as compared to control plant [23]. Another study reported that Fe-based MNOs also significantly increased the concentration of ABA (223%) and SA (292%) at 100 µg/mL in tobacco leaf under the TMV infection as compared to the control plant [21]. Subsequently, our findings have demonstrated that the application of C_60_-based MNOs at 100 mg/L can suppress CCYV infection by modulating the concentrations of plant hormones that are related to defense. Certainly, further research is needed to understand the basis of the viral infection and MNOs prompted plant phytohormone response to enhance this imperative disease management approach.

### 3.3. Effect of MNOs on the Photosynthetic Activity of N. benthamiana 

**Figure 4:**
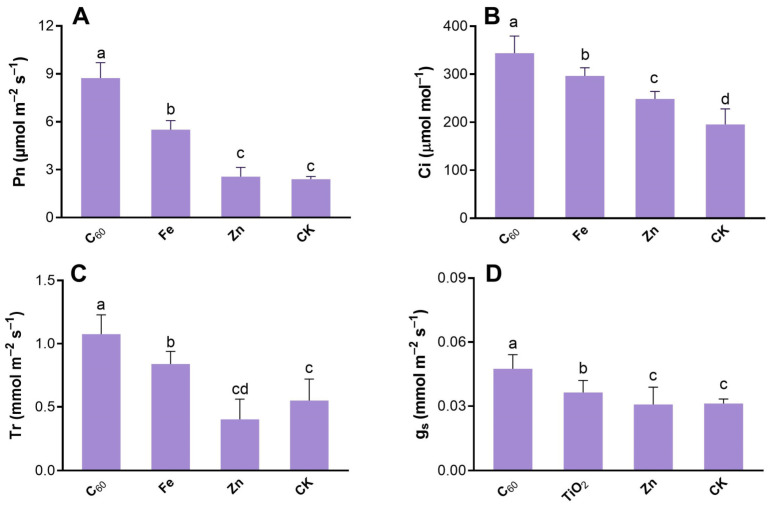
Carbon and metal-based MNOs significantly enhanced (**A**) net photosynthesis rate (Pn), (**B**) concentration of intercellular CO_2_ (Ci), (**C**) transpiration rate (Tr), and (**D**) stomatal conductance (gs). Various letters represent the significance level across the applied treatments (*p* < 0.05) (n = 3).

The photosynthetic performance of tobacco leaves treated carbon and metal-based MNOs was assessed in response to the infection of CCYV. Foliar application of carbon-based MNOs at 100 mg/L significantly increased Pn nearly 5-fold as respective to the control plants. With metal-based MNOs (Fe and Zn) at 100 mg/L concentration, the rate of Pn were 129% and 7% increased than that in control plants (Figure 4A). Additionally, the lowest increment in Pn was noted under the application of Zn-based MNOs as compared to the control. 

The photosynthetic activity, which is assessed by the entry of CO_2_ through the stomatal cell and fixation within the chloroplast, can be changed when plants are under stress [50]. Additionally, Zhang et al. have reported that tomato plants exposed to higher CO_2_ concentrations show a decrease in the severity and incidence of TMV infection [51]. Moreover, C_60_-based MNOs at 100 mg/L considerably enhanced the transpiration rate by 95%, stomatal conductance by 52%, and intercellular CO_2_ by 75% as respective to the control (Figure 4B–D). Conversely, the application of Zn-based MNOs at 100 mg/L enhanced Ci by 27% compared to control plants (Figure 4D) as well as significantly decreased the transpiration rate and Gs than that in control plants. The results of the current study are in agreement with a previous study in which both CNTs and C_60_-based MNOs significantly increased (4 folds) the Pn at 100 and 200 mg/L under the inoculation of TMV as compared to the control plants [23]. Another study has documented that CNTs enhanced the photosynthesis activity in plants (soybean, barley, and corn) [38]. Recently, a study reports that foliar application of Fe3O4-based MNOs to *Pseudostellaria heterophylla* significantly increased the Pn (30%) by promoting the chlorophyll content [52]. The enhancement of chlorophyll content is related to the pigment and photosynthetic gene that further enhanced the inoculation of Fe_3_O_4_ MNOs to barley (*Hordeum vulgare* L.) [53]. Yoon et al. have reported that Fe-based NPs (nZVI) significantly enhanced the photosynthetic performance, which ultimately increased the plant biomass and promoted the CO_2_ uptake in *Arabidopsis thaliana* [54]. 

**Figure 5:**
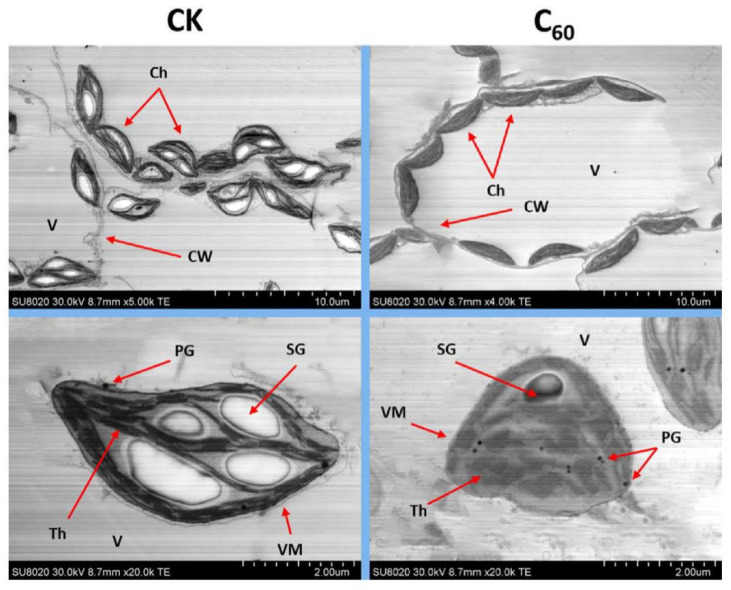
TEM observation of *N. benthamiana* leaf tissues that were infected by CCYV: GFP after foliar application of different MNOs at 100 mg/L. Ch, chloroplast; CW, cell wall; V, vacuole PG, plastoglobule; SG, starch granule, Th, thylakoid membraneand VM, vacuole membrane.

High performance of photosynthetic activity increased by MNOs that enhanced the overall growth and development of plants which finally resulted in enhanced crop yield [18]. Furthermore, Kromdijk et al. have documented that higher induction of CO_2_ in leaves also leads to increased dry matter ~15% in tobacco (*N. benthamiana*) [55]. In the current study, found that higher fresh biomass of the plants within C_60_-based MNOs treatment (Figure 2A) as compared to other applied MNOs. It is possible that the enhanced photosynthesis induced by C_60_-based MNOs application is due to the normal ultra-structure and integrity of the chloroplasts that were maintained in the CCYV-infected tobacco leaves. The above results were verified by TEM observation of the chloroplast ultrastructures of the leaf (Figure 5). 

### 3.4. Ultrastructural Analysis of Leaf Chloroplasts Infected by CCYV

In plant cells, the chloroplast is one of the important dynamic organelles. It performs photosynthesis, productions of main phytohormones, plays a crucial role in the plant defense response as well as is crucial for inter-organelle signaling [56]. As shown in Figure 5, the chloroplast images of plants that were exposed to 100 mg/L C_60_-based MNOs. For the chloroplast observation, we selected the systemic leaves with the same growth stage and position. The TEM observation indicated that infection of CCYV remarkable damaged the cellular integrity of tobacco leaves, as shown by the development of abnormally distributed plastoglobulus, multiple large starch globules, distorted thylakoid membranes, invagination of vesicular membranes and disarranged grana stalks (Figure 4A, upper panel). Moreover, the number of chloroplasts was lower than that of control plant leaves. Similar abnormal changes were reported in the tissues of Fe and Zn-based MNOs treatments, showing the unhindered negative effects of viral infection. Whereas, ultrastructure observation of CH in C_60_-based MNOs treated leaves were significantly lesser damaged as compared to control and other applied MNOs. As well as the chloroplasts number was slightly (but non-significant) downregulated in both treatments (Fe and Zn-based MNOs); which implies that overall, there was no effect on the structural integrity of the organelle. These TEM observations concluded that the harmful impacts of CCYV infection in plant chloroplasts were significantly lesser or stopped by the use of C_60_-based MNOs as a pre-application. This suppression of damage in the new leaf tissues obviously indicated an improved plant growth as opposed to the viral pathogen. Previous studies have reported that the virus caused ultrastructural and functional damages/changes in chloroplast tissues in numerous plant species [57]. The chloroplasts are the well-known main targets of viruses that utilize these organelles for replication and pathogenesis [56,57]. Our results are in agreement with a recently reported study in which CNTs and C_60_-based MNOs prevented the chloroplast tissues from the infection of TMV in tobacco plants than that control plants at 100 and 200 mg/L concentrations [23]. Subsequently, we conclude that pretreatment with C_60_-based MNOs highly stimulates the plant defense system as opposed to the viral infection by minimizing/delaying the damage and building up the plant immunity averse to the pathogen.

### 3.5. Carbon and Metal-Based MNOs Treatments Suppressed the CCYV Infection 

**Figure 6:**
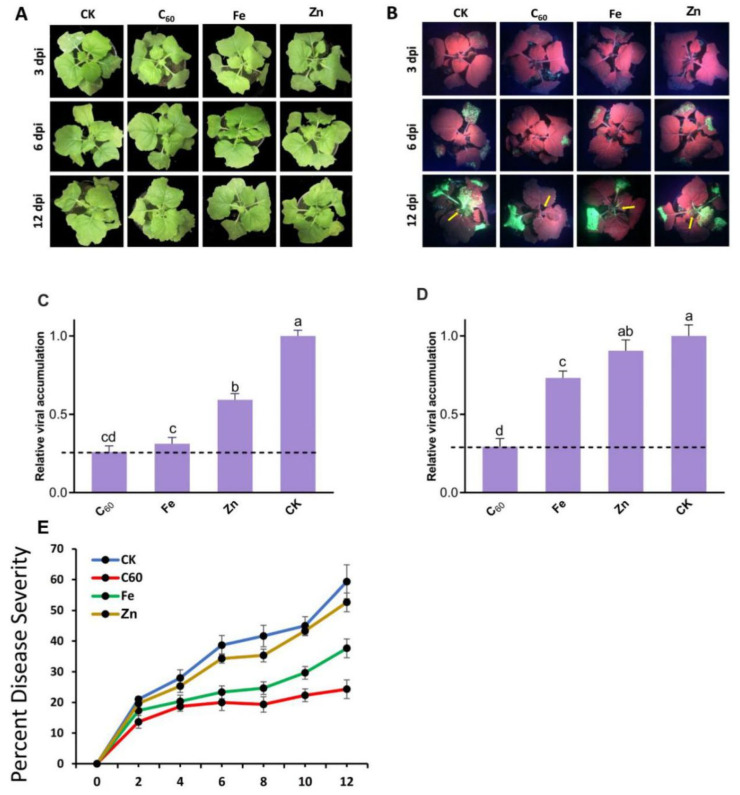
Effect of carbon and metal-based MNOs on the inhibition of CCYV multiplication in *N. benthamiana*. RT-qPCR was done to estimate the relative accumulation of viral coat protein mRNA. (**A**) CCYV infection (observed by GFP fluorescence) in the tobacco leaves with various treatments, (**B**) disease index of the CCYV infection of the tobacco plants, (**C**) relative accumulation of viral coat protein mRNA, (**D**) GFP mRNA in the *N. benthamiana* leaves and (**E**) percentage of disease severity in leaves (*p* < 0.05) (n = 3). Lowercase letters are Nanoparticle formula. Fullerene (C60).

Nanoparticles play important role in enhancing the growth and development of plants as well as promoting tolerance against biotic/abiotic stress [58,59]. Lately, it has been reported that various kinds of NMs used as anti-pathogenic agents play significant roles in disease prevention [20,22]. The physiological effects of MNOs treated *N. benthamiana* to CCYV infections were remarkably noted and photographed at 3, 6, and 12 dpi for better observation. However, the qualitative effects of virus inoculation and symptoms development were apparent as early as 3 dpi, the infection was not observable among the NMs treated plants. In plants treated with C_60_-based MNOs a normal growth was reported compared to the infected controls and the systemic leaves at 12 dpi, showed no symptoms of CCYV infection (Figure 6B). The disperse of viral infection from inoculated leaves to the newly emerged leaves was prohibited by foliar application of C_60_-based MNOs. Although, Fe and Zn-based MNOs did not repress viral infection or symptoms as compared to the control. For the detailed mechanism by which MNOs suppress viral disease development, we measured the expression levels of CCYV-CP in the systemic leaves at 5 dpi. The results clearly demonstrated that the abundance of viral CP transcripts was significantly reduced by 4 fold in the C_60_-treated plants (100 mg/L) as respective to the virus-infected control plants, respectively (Figure 3). Additionally, both Fe and Zn-based MNOs treatments had slightly reduced the mRNA concentration of CCYV-CP, proposing limited positive impacts but the effect was not as strong as with the carbon-based exposure. The suppression of viral CP by C_60_ indicates that these NMs interfere with CCYV replication and subsequently inhibited the viral movement into apical tissues. Current results are similar to a recently reported study in which C_60_-based NPs significantly suppressed the viral infection as respective to the control plant [23]. Cai et al. have documented that Fe-based NPs enhanced plant immunity against TMV [21]. Zn-based NPs also play an important role to protect the tobacco plants against TMV [22].

Unsurprisingly, the GFP quantification shows a remarkable viral accumulation in the inoculated tobacco leaves at the site of infection. The presence of CCYV in the systemic leaves was measured by GFP signal intensity imaging at 3, 5, and 12 dpi (Figure 6A,B). Notably, treatment with C_60_-based MNOs at 100 mg/L significantly reduced viral infection. The fresh emerging systemic leaves had negligible fluorescence intensity, representing that the virus was incapable to establish successful infection in these tissues. Otherwise, Fe and Zn application had a slight impact on the development of viral symptoms. These quantifications were further confirmed by observing the relative expression of GFP transcripts in systemic leaves; analysis exposed that 100 mg/L C_60_-based MNOs treatments downregulated the GFP expression by 241% more than that in control plants (Figure 6d). Briefly, GFP imaging, phenotypic observation, and RT-qPCR investigation clearly proved that C_60_ at 100 mg/L was significantly highly effective at repressing the growth of viral symptoms and at preventing CCYV propagation into systemic leaves. Further comprehensive research was then required at understanding the mechanistic in-depth processes and the protective roles of MNOs during plant and disease development.

## 4. Conclusions

The current research emphasizes on antiviral effects of carbon-based NMs against CCYV-infected *N. benthamiana* host plants. Interestingly, we found that at 100 mg/L concentration, the carbon-based NMs not only significantly reduced the appearance of viral disease symptoms but also, the relative abundance of viral particles in the newly emerged leaf tissues was remarkably inhibited. The downregulation of viral CP transcripts and mRNA of GFP was obvious in the systemic tissues of CCYV-infected plants. This is perhaps because of the possible disturbance of the inter-cellular viral movement and hindered replication events caused by carbon-based NMs. It is a well-known fact that chloroplast is the common target of plant viruses during plant-virus interactions and conversely, plants do use chloroplast-based defense weapons to combat viruses. Our data involving chloroplast ultrastructural observation and measurements of biochemical parameters unequivocally demonstrates that treatment of carbon-based NMs greatly imposed a protective and antiviral effect during CCYV-plant interaction. As a result, not only the chloroplast structural integrity of the *N. benthamiana* was retained but also, the structural and functional properties of the photosynthetic machinery remained unaffected. This data supports our hypothesis that during plant-virus interactions, carbon-based NMs interfere with the molecular events (more specifically molecular interactions between plant and viral factors) that abort the establishment of successful viral infection, ultimately resulting in no/reduced disease symptoms and viral particles. Categorically, the results of our experimental data shed light on the use of carbon-based NMs to protect the plants against CCYV infection and to enhance the overall growth and development by augmenting the disease resistance of the host plants. To our knowledge, this is the first study explaining the combinatorial impact of NMs and viral infection on the *N. benthamiana* host plants. These findings will enrich our understanding of efforts to develop environmental-friendly NMs-based antiviral medicines to cure/protect the plants.

## Figures and Tables

**Table 1:** Properties of carbon and metal-based MNOs.

Parameter	C_60_	Fe	Zn
Size (nm)	50 ± 5	20–30	30 ± 7
Zeta Potential (mV)	−10.8 ± 0.8	22 ± 4.1	−14 ± 0.5
DLS (nm)	882 ± 85	127 ± 3.5	63.92 ± 6.21

## Data Availability

Not applicable.

## Appendix A

**Table A1:** Primers used for RT-qPCR analysis.

Primer	Sequence (5′-3′)	Product Size
CCYV-CP-F	CTGAGGATGGAGCCGAAG	200 bp
CCYV-CP-R	TCCCTCATACACTGTTCG	
GAPDH-F1	GTTGCCTTCCAAACCTCT	200 bp
GAPDH-R1	TTGAGAAGGTGAGAGGCT	
GFP-F	ACTACTGGAAAACTACCTG	200 bp
GFP-R	TCAAACTTGACTTCAGCAC	
